# Supplementary material for: One year cross-sectional study in adult and neonatal intensive care units reveals the bacterial and antimicrobial resistance genes profiles in patients and hospital surfaces
Source: PLoS One. 2020 Jun 3;15(6):e0234127. doi: 10.1371/journal.pone.0234127 (PMC7269242; doi:10.1371/journal.pone.0234127)
Supplement: S1 Fig — (PDF) [file pone.0234127.s001.pdf]

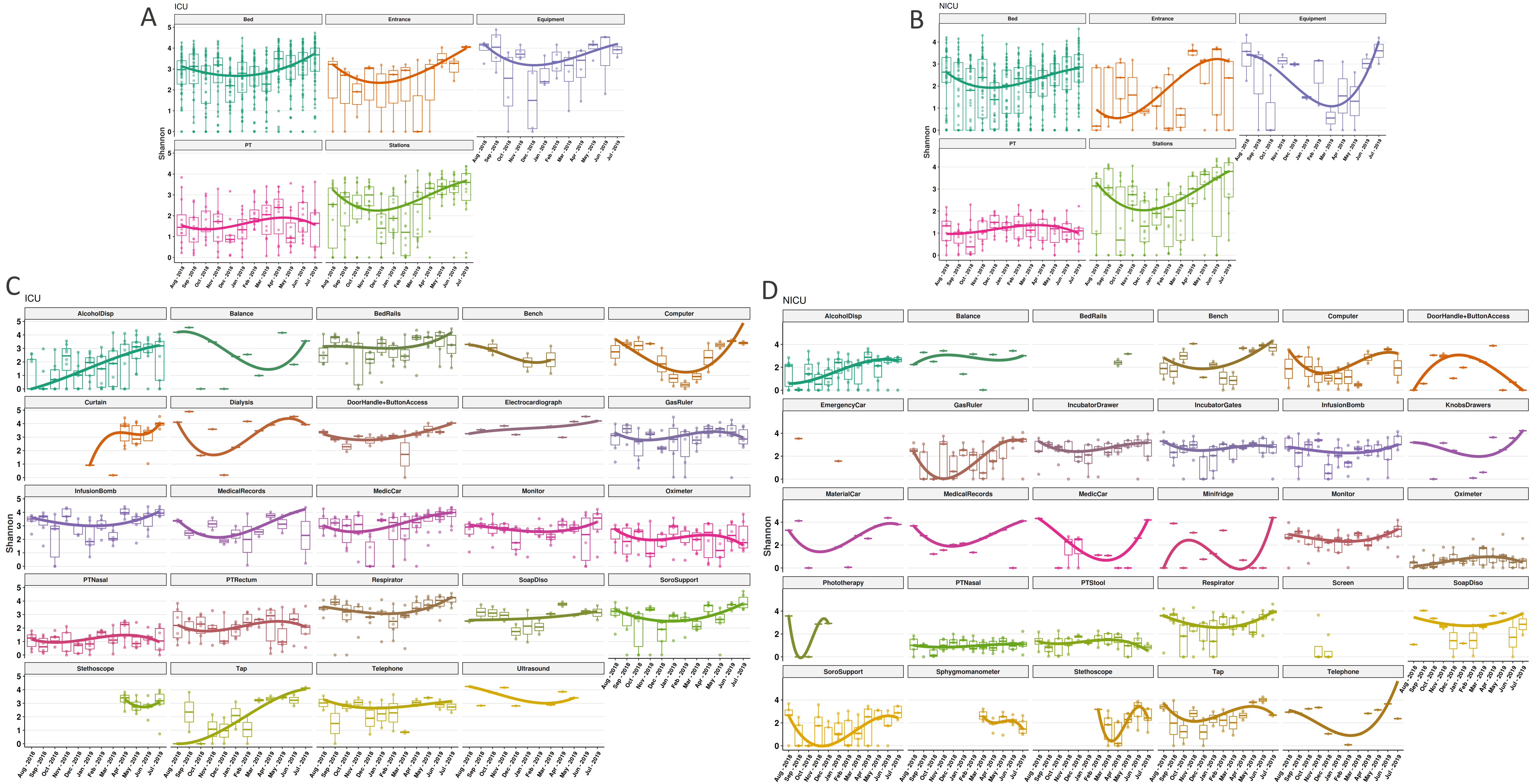

S1 Fig. Shannon diversity index profiles considering all samples from **(A)** ICU and **(B)** NICU in each month of analysis and sample collection locations: Bed, Entrance, Equipment, Patient (PT) and Stations. Shannon index for specific site collection in each intensive care unit **(C)** ICU and **(D)** NICU in each month. Boxplots were represented by median values and with a trend line for diversity index values over the year.
