# Supplementary material for: One year cross-sectional study in adult and neonatal intensive care units reveals the bacterial and antimicrobial resistance genes profiles in patients and hospital surfaces
Source: PLoS One. 2020 Jun 3;15(6):e0234127. doi: 10.1371/journal.pone.0234127 (PMC7269242; doi:10.1371/journal.pone.0234127)

S2 Fig. Weighted UniFrac Beta-diversity PCoA plots for all samples collected in both units, ICU and NICU separated in three different timelines for better visualization, **(A)** from Aug-2018 to Nov-2018, **(B)** Dez-2018 to Feb-2019 and **(C)** Apr-2019 to Jul-2019.

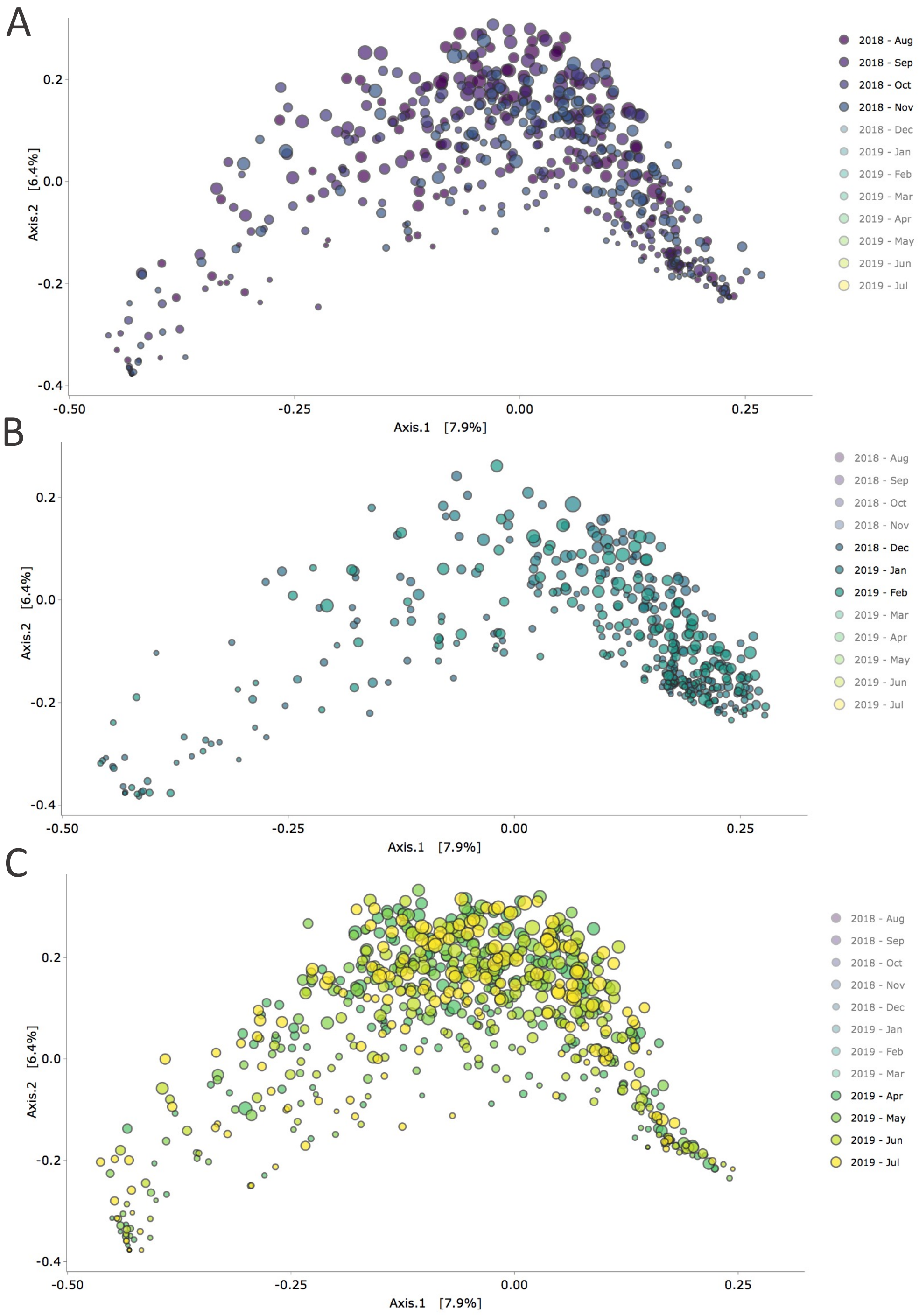

Supplement: S2 Fig — (PDF) [file pone.0234127.s002.pdf]
