# Supplementary material for: One year cross-sectional study in adult and neonatal intensive care units reveals the bacterial and antimicrobial resistance genes profiles in patients and hospital surfaces
Source: PLoS One. 2020 Jun 3;15(6):e0234127. doi: 10.1371/journal.pone.0234127 (PMC7269242; doi:10.1371/journal.pone.0234127)
Supplement: S3 Fig — (PDF) [file pone.0234127.s003.pdf]

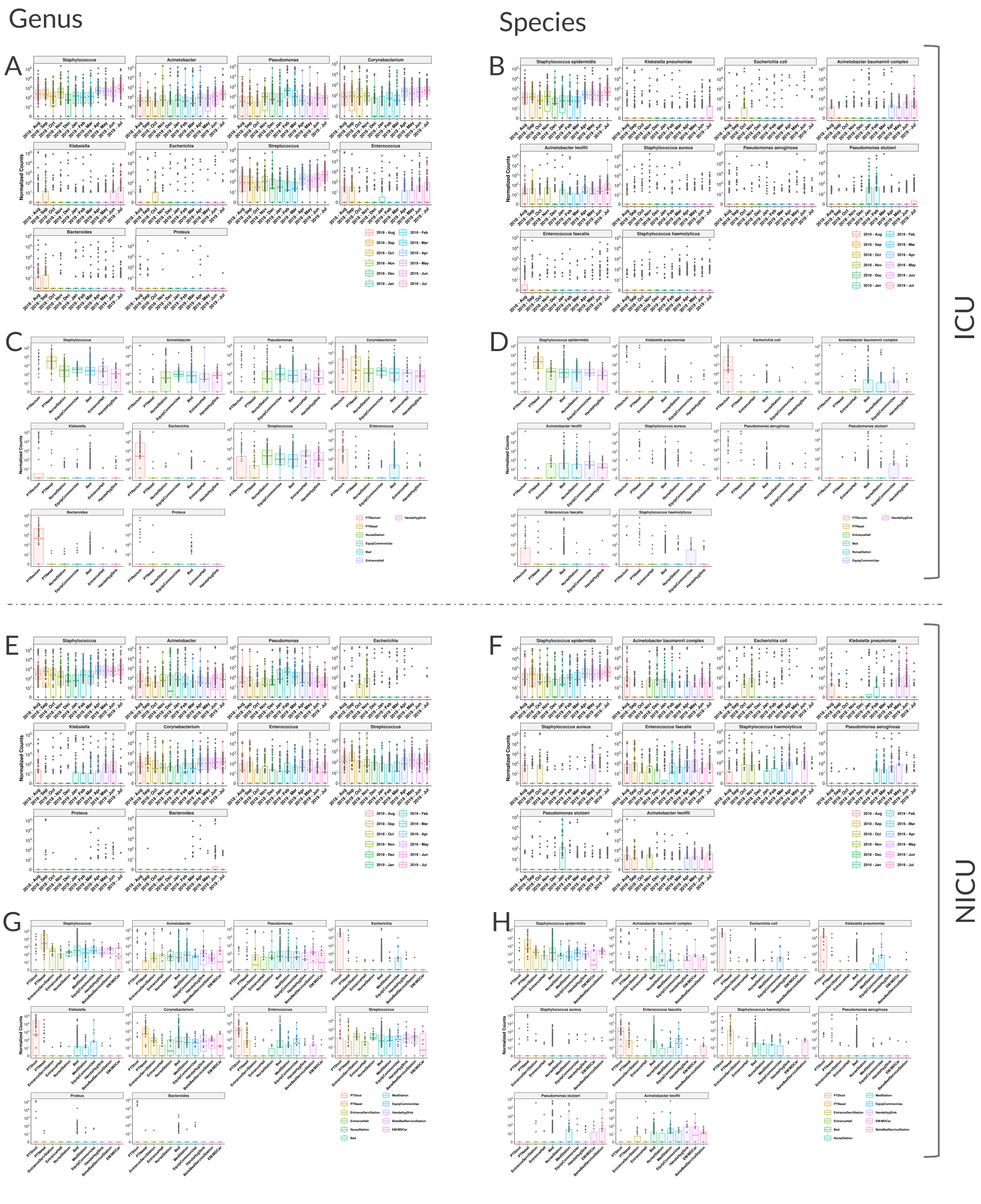

S3 Fig. Most abundant bacteria detected and classified in Genus and Species. ICU abundant bacteria by month **(A-B)** and by sample location **(C-D)**. NICU abundant bacteria by month **(E-F)** and by sample location **(G-H)**.
