# Supplementary material for: One year cross-sectional study in adult and neonatal intensive care units reveals the bacterial and antimicrobial resistance genes profiles in patients and hospital surfaces
Source: PLoS One. 2020 Jun 3;15(6):e0234127. doi: 10.1371/journal.pone.0234127 (PMC7269242; doi:10.1371/journal.pone.0234127)
Supplement: S4 Fig — (PDF) [file pone.0234127.s004.pdf]

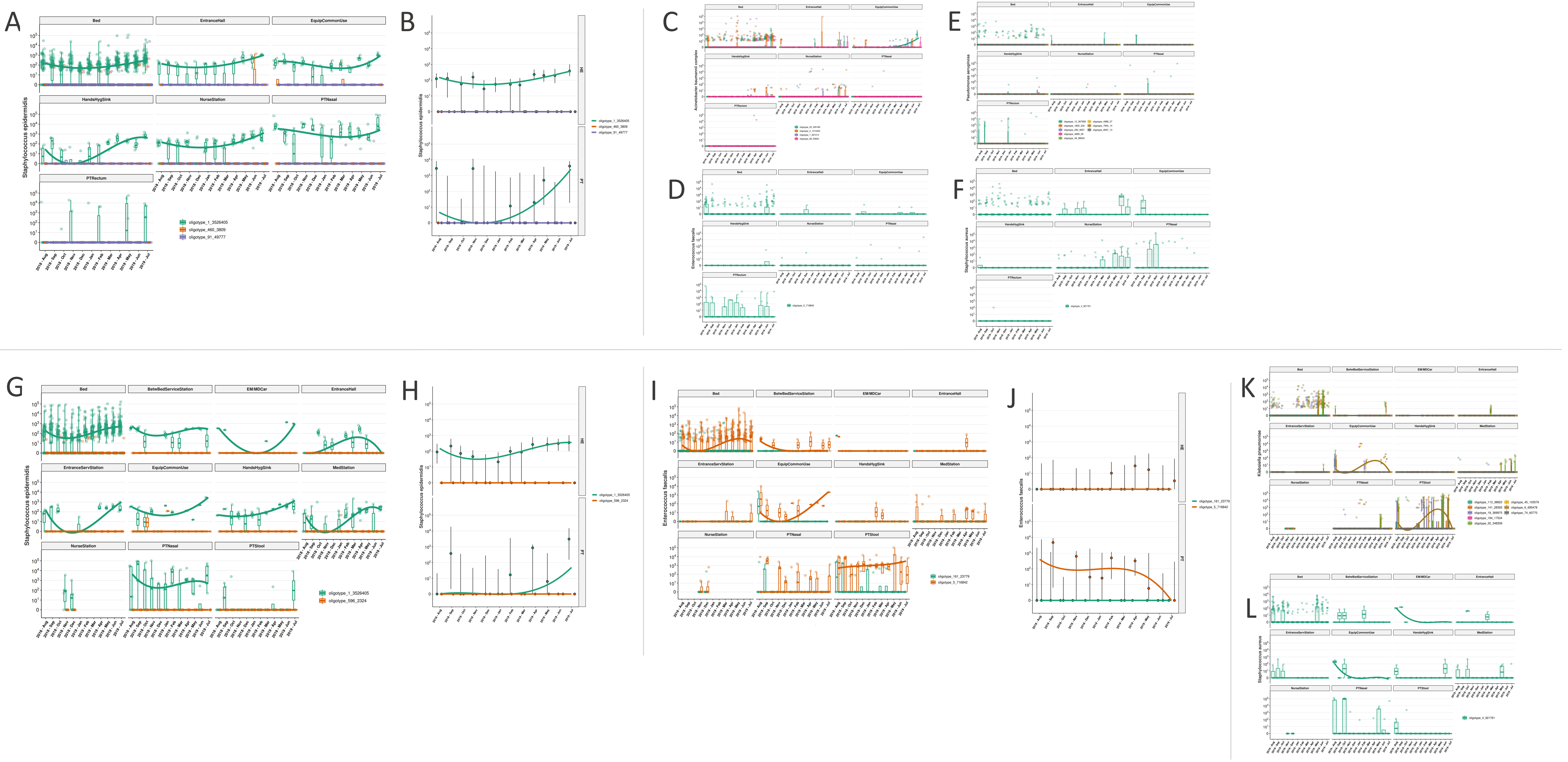


**S4 Fig.** Longitudinal profile of specific bacteria and its most abundant oligotypes found in (**A-F**) ICU and (**G-L**) NICU locations. (**A**) *S. epidermidis* oligotype abundances in different locations and separated (**B**) in patients (PT) and environmental samples (HE) over the year in the ICU. (**C**) *A. baumannii* complex (**D**) *E. faecalis* (**E**) *P. aeruginosa* and (**F**) *S. aureus* oligotypes longitudinal abundance profiles in sample collection locations from ICU. (**G**) *S. epidermidis* oligotypes distributions along the year in NICU locations and their longitudinal profile in (**H**) patients and environmental samples. (**I**) *E. faecalis* oligotypes abundances in collected locations and their distributions in (**J**) patients and environmental samples. (**K**) *K. pneumoniae* and (**L**) *S. aureus* oligotypes abundance profiles in NICU locations over the year analyzed. A trend line was added in the boxplots to better visualize the longitudinal profiles over the year.
