# Supplementary material for: One year cross-sectional study in adult and neonatal intensive care units reveals the bacterial and antimicrobial resistance genes profiles in patients and hospital surfaces
Source: PLoS One. 2020 Jun 3;15(6):e0234127. doi: 10.1371/journal.pone.0234127 (PMC7269242; doi:10.1371/journal.pone.0234127)
Supplement: S5 Fig — (PDF) [file pone.0234127.s005.pdf]

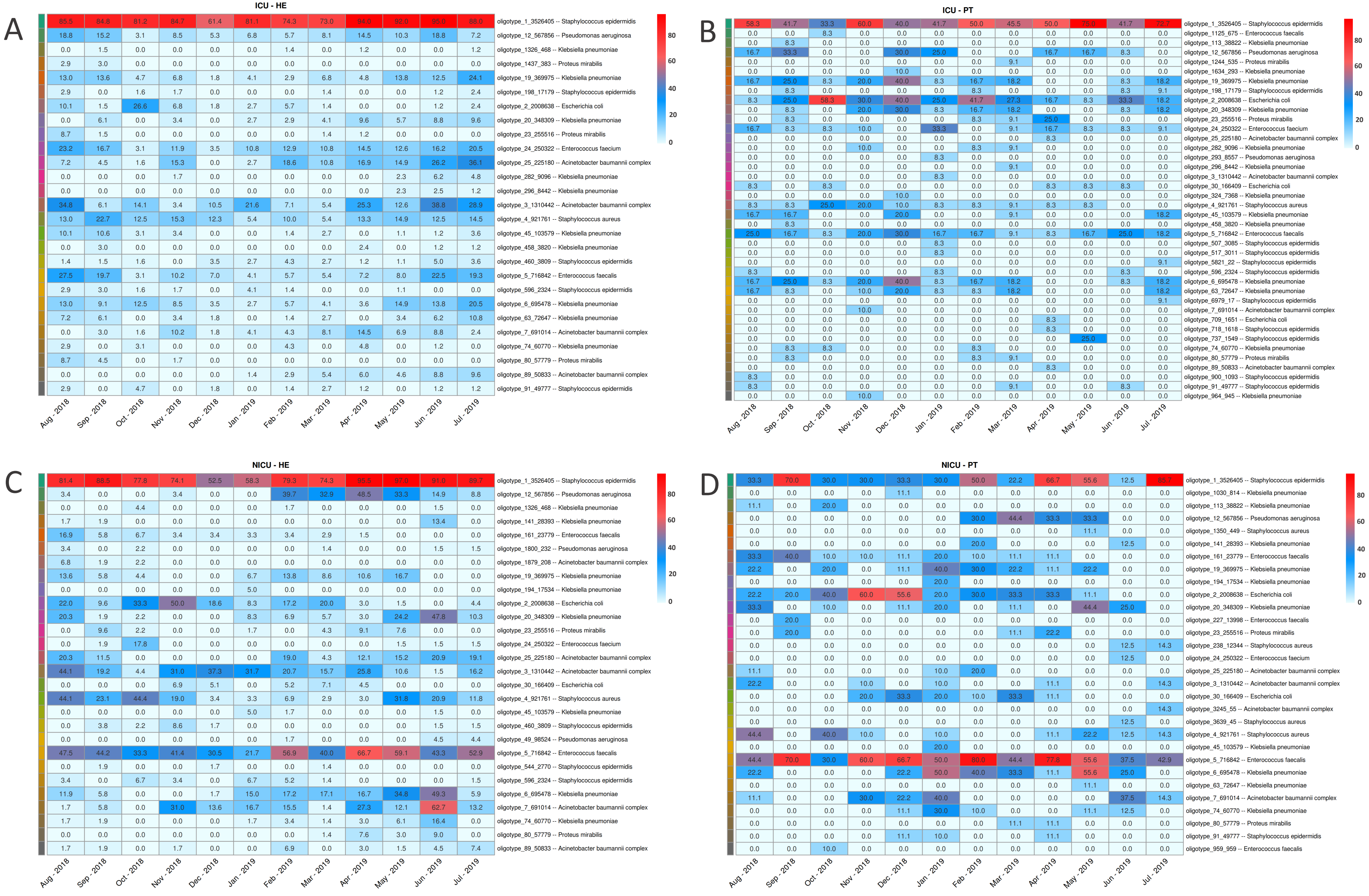

S5 Fig. Proportion of positive samples for the most abundant oligotypes in (**A**) ICU environment, (**B**) ICU patients, (**C**) NICU environment and (**D**) NICU patients. Oligotypes are identified by a unique code, along with its count number (total reads detected along the analysis) - Oligotype_code_count, and the bacterial taxonomy attributed by the bioinformatics pipeline. Color scales represents the proportion of positivity in the group of samples.
