# Supplementary material for: One year cross-sectional study in adult and neonatal intensive care units reveals the bacterial and antimicrobial resistance genes profiles in patients and hospital surfaces
Source: PLoS One. 2020 Jun 3;15(6):e0234127. doi: 10.1371/journal.pone.0234127 (PMC7269242; doi:10.1371/journal.pone.0234127)
Supplement: S6 Fig — (PDF) [file pone.0234127.s006.pdf]

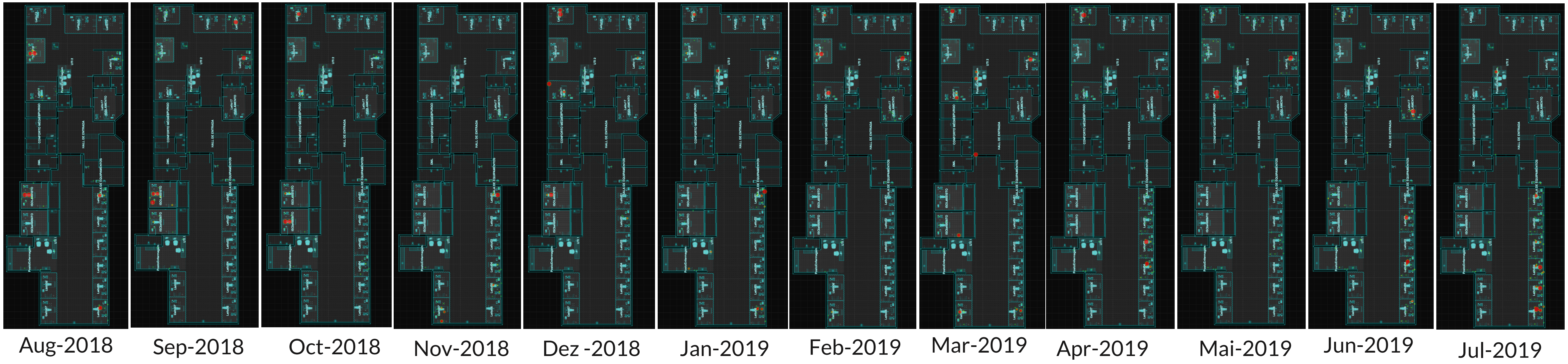

S6 Fig. Risk map ICU. The blueprint of hospital ICU was used to plot each month of analysis in a timely way to observe the bacterial contamination in the ICU over the year. Red spots represent the bacterial density (total number of reads) detected. Only bacterial sequences from the HAIrB group were plotted in this map. Following each month map it could be observed that in the ICU the red spots spots were not consistently in the same locations, they vary, and their intensities were relatively low.
