# Supplementary material for: One year cross-sectional study in adult and neonatal intensive care units reveals the bacterial and antimicrobial resistance genes profiles in patients and hospital surfaces
Source: PLoS One. 2020 Jun 3;15(6):e0234127. doi: 10.1371/journal.pone.0234127 (PMC7269242; doi:10.1371/journal.pone.0234127)
Supplement: S7 Fig — (PDF) [file pone.0234127.s007.pdf]

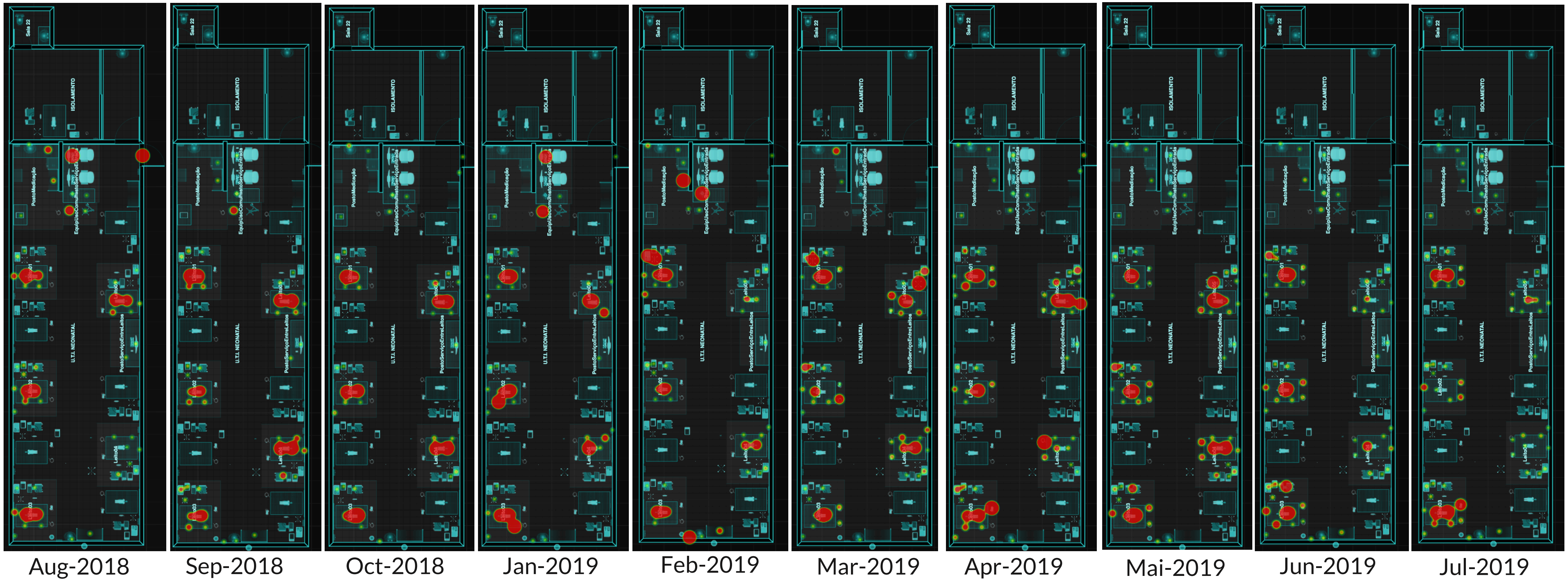

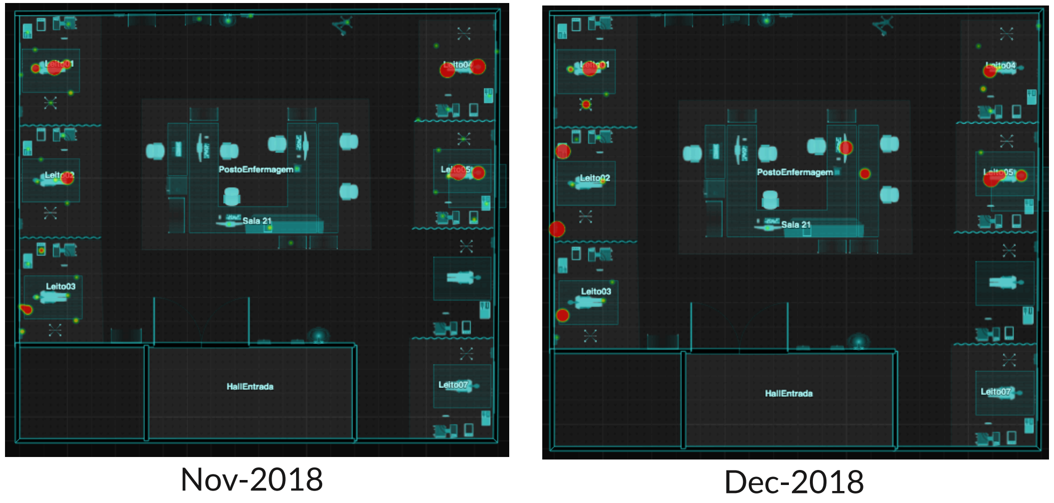

S7 Fig. Risk map NICU. The blueprint of hospital NICU was used to plot each month of analysis in a timely way to observe the bacterial contamination in the ICU over the year. Red spots represent the bacterial density (total number of reads) detected. Only bacterial sequences from the HAIrB group were plotted in this map. November and December are separated in different maps since interventions were performed and the NICU changed locations during this two months. Following each month in the map, it could be observed that in the NICU the red spots of each month have consistent high and widely spread intensities.
