# Supplementary material for: One year cross-sectional study in adult and neonatal intensive care units reveals the bacterial and antimicrobial resistance genes profiles in patients and hospital surfaces
Source: PLoS One. 2020 Jun 3;15(6):e0234127. doi: 10.1371/journal.pone.0234127 (PMC7269242; doi:10.1371/journal.pone.0234127)
Supplement: S1 Table — (PDF) [file pone.0234127.s008.pdf]

Supplementary table 1. Sample collection sites

Source	Locations	Specific Sites	ICU Samples	NICU Samples
Environment	Equipment Common Use	Balance	12	12
Environment	Medical and Nurse Stations	Bench	10	22
Environment	Bed	InfusionBomb	69	59
Environment	Equipment Common Use	MedicCar	83	14
Environment	Bed / Medical and Nurse Stations	MaterialCar	-	10
Environment	Nurse Stations and Entrance Stations	Computer	24	24
Environment	Equipment Common Use	Dialysis	12	-
Environment	Bed / Medical and Nurse Stations / Entrance Hall	AlcoholDisp	106	76
Environment	Medical and Nurse Stations	SoapDisp	24	19
Environment	Equipment Common Use	Electrocardiograph	12	-
Environment	Equipment Common Use	Phototherapy	-	5
Environment	Medical Station	Minifridge	-	12
Environment	Bed	BedRails	70	3
Environment	Bed	IncubatorDrawer	-	58
Environment	Bed	IncubatorGates	-	58
Environment	Entrance Hall	DoorHandle+ButtonAccess	24	13
Environment	Bed	Monitor (Cardiac)	70	60
Patient	Nasal	PTNasal	70	59
Patient	Stool	PTStool	-	52
Patient	Rectal	PTRectum	68	-
Environment	Bed	Oximeter	70	60
Environment	Nurse Station	MedicalRecords	24	12
Environment	Service Station Between Beds	KnobsDrawers	-	12
Environment	Bed	GasRuler	70	60
Environment	Bed	Respirator	70	60
Environment	Bed	IVStand	70	59
Environment	Nurse Station / Entrance Station	Telephone	24	12
Environment	Medical Station / Hands Hygiene Sink	Tap	24	24
Environment	Equipment Common Use	Ultrasound	9	-
Environment	Equipment Common Use	EmergencyCar	-	2
Environment	Bed	Screen	-	8
Environment	Bed	Curtain	23	-
Environment	Bed	Stethoscope	24	26
Environment	Bed	Sphygmomanometer	-	25
