# Supplementary material for: One year cross-sectional study in adult and neonatal intensive care units reveals the bacterial and antimicrobial resistance genes profiles in patients and hospital surfaces
Source: PLoS One. 2020 Jun 3;15(6):e0234127. doi: 10.1371/journal.pone.0234127 (PMC7269242; doi:10.1371/journal.pone.0234127)
Supplement: S2 Table — (PDF) [file pone.0234127.s009.pdf]

Supplementary table 2. Monthly collected samples and sequencing information.

Year	Month	Total Samples*	Seq Run ID	MiSeq Kit	MiSeq Kit Lot	Total Sequenced reads/Kit**	Mean Sample Coverage*	PhiX Error Rate	Clusters Passing Filter
2018	August	162	BVT63	V2-300	20259348<br>20271993	14,099,567	30,847	1.8 %	93 %
2018	September	163	BWF3K	V2-300	20265835<br>20271993	13,685,913	27,903	2.6 %	94 %
2018	October	161	BWF45	V2-300	20265835<br>20271993	13,908,897	30,079	3.4 %	94 %
2018	November	156	C3H22	V2-300	20282166<br>20287469	15,110,301	33,113	3.4 %	93 %
2018	December	155	C4GW4	V3-600	20286539<br>20298973	23,772,082	27,136	2.2 %	91 %
2019	January	162	C4KLK	V2-500	20286530<br>20295366	15,070,932	25,398	1.4 %	93 %
2019	February	163	C59HL	V2-300	20294299<br>20310032	17,036,891	31,176	2.0 %	92 %
2019	March	171	C68TC	V2-500	20298691<br>20311201	14,646,656	30,505	1.5 %	93 %
2019	April	181	C6YT5	V2-300	20306438<br>20330866	13,411,201	31,086	1.9 %	94 %
2019	May	183	C7L32	V2-300	20306438<br>20316702	14,625,019	30,222	2.1 %	93 %
2019	June	179	CFD4C	V2-300	20343603<br>20320219	16,369,195	37,163	2.2 %	92 %
2019	July	178	CH3HT	V2-300	20351939<br>20358460	16,756,668	36,677	2.5 %	92 %

* Considering only samples from this project.

** The sequencing kit may contain other samples, not just the ones included in this project.
