# Supplementary material for: One year cross-sectional study in adult and neonatal intensive care units reveals the bacterial and antimicrobial resistance genes profiles in patients and hospital surfaces
Source: PLoS One. 2020 Jun 3;15(6):e0234127. doi: 10.1371/journal.pone.0234127 (PMC7269242; doi:10.1371/journal.pone.0234127)
Supplement: Attachment — Submitted filename: PLoS One _Response to reviewers.docx [file pone.0234127.s010.docx]

Journal requirements:

When submitting your revision, we need you to address these additional requirements:

1.    Please ensure that your manuscript meets PLOS ONE's style requirements, including those for file naming. The PLOS ONE style templates can be found at <http://www.plosone.org/attachments/PLOSOne_formatting_sample_main_body.pdf> and <http://www.plosone.org/attachments/PLOSOne_formatting_sample_title_authors_affiliations.pdf>

RESPONSE: We revised the style requirements and necessary corrections were made according to PLOS ONE manuscript body formatting guidelines (February 2020).

2. Thank you for stating the following in the Competing Interests section:

I have read the journal's policy and the authors of this manuscript have the following competing interests: AC, AS, GC, DB, VS, LO are currently full-time employees of BiomeHub (SC, Brazil), a research and consulting company specialized in microbiome technologies.

Please confirm that this does not alter your adherence to all PLOS ONE policies on sharing data and materials, by including the following statement: "This does not alter our adherence to  PLOS ONE policies on sharing data and materials.” (as detailed online in our guide for authors http://journals.plos.org/plosone/s/competing-interests).  If there are restrictions on sharing of data and/or materials, please state these. Please note that we cannot proceed with consideration of your article until this information has been declared.

Please include your updated Competing Interests statement in your cover letter; we will change the online submission form on your behalf.

Please know it is PLOS ONE policy for corresponding authors to declare, on behalf of all authors, all potential competing interests for the purposes of transparency. PLOS defines a competing interest as anything that interferes with, or could reasonably be perceived as interfering with, the full and objective presentation, peer review, editorial decision-making, or publication of research or non-research articles submitted to one of the journals. Competing interests can be financial or non-financial, professional, or personal. Competing interests can arise in relationship to an organization or another person. Please follow this link to our website for more details on competing interests: http://journals.plos.org/plosone/s/competing-interests

RESPONSE: We confirmed that the competing interests does not alter our adherence to all PLOS ONE policies and performed the requested updates in the cover letter with the following statement - I have read the journal's policy and the authors of this manuscript have the following competing interests: AC, AS, GC, DB, VS, LO are currently full-time employees of BiomeHub (SC, Brazil), a research and consulting company specialized in microbiome technologies. This does not alter our adherence to PLOS ONE policies on sharing data and materials. All authors have read and approved the manuscript.

Response to reviewers

We thank to the reviewers for the significative contributions in revising this manuscript. All the questions and suggestions helped to improve the manuscript quality. Responses for each point were addressed below.

Reviewer #1: Overall comments: The English language could be reviewed and improved throughout the document, mainly with some sentence constructions.

RESPONSE: The manuscript was revised and sentences rephrased to improve the English language.

The figure legends as well as legends for supplementary files need to be more detailed, referring to various aspects of the figure components.
RESPONSE: All figure legends were revised and improved.

Line 79-82: I think this needs rewording, the message in the sentence seems to be lost.
RESPONSE: The sentence was revised and rewritten.

Lines 202-203: What were the positive controls used for the RT-PCR? And probe details? This information could be useful and should be included in the supplementary information.
RESPONSE: In fact, there is no RT-PCR assay in the manuscript. The Real-Time PCR was performed based on bacterial genomic DNA, without reverse transcription (RT). Probe details were already included in the methods section. We added an statement about positive controls, along with an acknowledgement for the Prof. Dr. Ana Gales, that kindly give us the positive controls strains.

Line 209: What was the criteria used to select isolates for genome sequencing? This information needs to be elaborated in the methods.
RESPONSE: We included an statement in the methods that bacterial isolates from the hospital routine surveillance swabs, during the same week that the molecular biology collection took place, were selected for whole genome sequencing.

Lines 252-255: Could the statistical significance for these differences be indicated in the figure and explained in the figure legend?
RESPONSE: Wilcoxon statistical test was performed and included in the figure. The statistical differences are indicated in the figure legend.

Lines 278-279: (Supp Fig 3) I appreciate that this is only the supplementary figure, but it is nevertheless almost incomprehensible. Maybe stick to only genus and species panels?
RESPONSE: Fig S3 was changed according to the suggestions.

Line 438: In Figure 7, it would be good if the ICU and NICU panels could be indicated for clarity in the figure
RESPONSE: Panels were indicated for ICU and NICU in Fig 7.

Lines 529-531: These sentences need to be reworded.
RESPONSE: The sentences were rewritten.

Line 557: A search shows the BioProject data to be currently unavailable. This needs to be addressed before this manuscriot can be accepted.

RESPONSE: BioProject PRJNA604445 was made available in the NCBI SRA repository <https://www.ncbi.nlm.nih.gov/bioproject/PRJNA604445>

Reviewer #2: Thank you for allowing me to review this very interesting and timely paper, One year cross-sectional study in adult and neonatal intensive care units reveals the bacterial and antimicrobial resistance genes profiles in patients and hospital surfaces. It is an important topic and this reviewer has a few comments.

Overall, the paper is written clearly. The introduction could provide the reader with more background on the infection issues in critical care units and mention the difference observed between the neonatal and adult ICUs.

RESPONSE: A new paragraph was included in the introduction section to clarify this.

The authors need to provide a reason and justification why they collected rectal swabs on adults and stool specimens on infants. These are two different microbiomes that the authors are attempting to compare.

RESPONSE: This experimental design was restricted by the hospital ethics committee, which didn’t allowed the collection of rectal swabs from the neonates in this project. Then we performed the best that we could do for obtaining the results, and defined the collection of freshly emitted stool from neonates. We now added this information on fresh stool in the methods section.

The authors need to provide the reader a summary of the neonatal and adult units that were the sites of the study-type of patients, tertiary hospital, etc.

RESPONSE: The information about the hospital (tertiary and public) were reinforced along the manuscript. There was only one ICU and one NICU unit in the hospital, the collection sites were the same for each month. Detailed sample collection sites were depicted in the S2 Table.

There are many figures in the main paper and many supplementary tables. Supplementary table 1 is very valuable in that it tells the reader the location of samples and the number of samples obtained from that area. This is an important table and perhaps needs to be in the main body of the paper.

RESPONSE: We included a table 1 in the methods section with a resumed view of the collection sites and number of samples.

Overall the figure and table legends need more information and need to define each abbreviation for the reader to interpret the figure/ table. For example in Table 1 first row, it is not clear what the purpose of this entry (*Isolated in the hospital previously the project period). Figure 2 is a great figure but it was difficult to decipher the color legends in C, D, and E. Again, the maps of the units (Supp. Figures 7&8) contain very valuable information and may help explain why some locations in the units are more contaminated than other however, the figure are difficult to read.

RESPONSE: All figure legends were modified and improved. Table 1 legend was altered, and the dubious information explained in the text. Figure 2 legend was fixed to improve comprehension of the colors and collection sites evaluated in each PCoA plot. In supplementary S7 and S8 figures the figure legends were improved but the main purpose of the maps is to give a wide view of the contamination status over the year, in a timely way, where we can see that in ICU the hotspots were in fewer locations varying each month but in NICU, they were constant in pretty much every month and location. It will be too complex to associate specific location for hotspots and the hospital blueprints in that figures. If they still difficult we can remove them.

The discussion mentions the nasal samples and the resistance genes that were identified but there is little mention in the paper of a comparison of nasal versus gut microbiome of the patients-resistant genes versus bacteria. A limitation paragraph might be helpful at the end of the discussion.

RESPONSE: The discussion was improved and such comparisons were included in the text.

Please double-space the manuscript.

RESPONSE: It was corrected along with other manuscript formatting requested by the journal guidelines.
